# Observations on the Ætiology of Bilious Fever

**Published:** 1830

**Authors:** John Cook Bennett

**Affiliations:** McConnelsville, Ohio


December, 1829.

---

Art. IV.—*Observations on the Ætiology of Bilious Fever.*—By Dr. John Cook Bennett, of McConnelsville, Ohio.

On the ætiology of autumnal fever, there remain some differences of opinion. I have no doubt, that marsh miasmata or putrid vegetable exhalations, are a fruitful cause of fever; but there seems to me much reason for believing that the putrid exhalation from dead aquatic animalculi, constitute a cause equally powerful. I propose to state a few facts which seem to me to go to the support of both opinions.

The water from the Zanesville canal dam, was let out, about the 20th of August, 1829. A short time before this, the mills of Mr. Granger, situated upon this canal, were destroyed by fire, and near 5000 bushels of grain, some of it partially burned, fell into the canal, and became very putrid. The water discharged from the canal, arrived at this place, [McConnelsville,] the next day. It was so offensive that horses would not drink it, and it could be distinctly smelt for several rods from the Muskingum. At the time the water arrived here, the wind blew from the west, and it continued in that direction several days. From the 24th to the 27th there were 10 new cases of fever, in the town, under the care of Dr. Barker, and about as many more the week following. Those under the care of the other physicians of the town, at the same time would add largely to the number. All these cases of bilious fever occured in the direction of the wind, from the offensive water. The inhabitants on the west side of the river, were very healthy at this time. But to return to Zanesville. Those who lived above the dam, of which I have spoken, or immediately adjacent to it, where the water was backed up, became sickly upon letting off the water, so as to expose a portion of the grain and other decayed vegetable matter; but upon shutting the gates and filling the dam again, with back water so as to cover those decaying substances, they recovered their health. These facts were communicated to me by Dr. S. A. Barker, a respectable physician of this place. They are interesting in several particulars.

1st. The putrid exhalations produced fever in about forty-eight hours after persons were exposed to their influence.

2nd. Those who lived about the dam, or back water, were healthy as long as the canal and dam were full; but as soon as it was emptied by letting off the water, they became sickly.

3rd. Those in this place, who were not in the direction of the wind, were quite healthy, while those who inhaled the effluvia and those upon whom the wind blew, were the subjects of the disease. Another fact.

I have been informed of another fact by a respectable traveller, that upon letting the water into the Ohio and Erie canal, this season, at and near Newark, the town and country were partially inundated--the cellars filled with water &c. in consequence of the banks of the canal being too porous to retain the water in its proper channel. The leaking of the canal banks produced a great quantity of stagnant water in its vicinity, and its usual concomitant followed--an unusual number of cases of bilious fever.

Now the question which naturally suggests itself is this-- What was the real cause of the sickness in the two cases related? I shall consider them under separate heads.

1st. The cause of the sickness at McConnelsville and Zanesville. This was evidently the putrid exhalations from the grain and other vegetable substances in the canal and dam. The water of this manufacturing canal, was so completely saturated with the miasmata, from the grain, that it remained sufficiently contaminated and offensive to produce fever when it arrived at McConnelsville—a distance of 27 miles. Now water being pure and incapable of generating disease of itself by any process whatsoever, the disease alluded to must have been produced by the putrefaction and decomposition of the grain, at Grainger's mills, which as I said fell into the canal at the burning of the Mill. This is a clear and satisfactory case of the production of bilious fever by miasmata or putrid vegetable exhalations.

2nd. The sickness at Newark, I am disposed to ascribe this to the death and decomposition of *animalculi*, rather than the decomposition of vegetable substances. Indeed, it seems to me, that this kind of decomposition as a source of autumnal fever, has been too much overlooked. Is not the green matter which covers the surface of stagnant water, the offspring of the putrefaction of animalculi? And may not the Hydrocyanic acid in a gaseous state, which Dr. Hempstead supposes to be the deleterious agent in the production of Autumnal fever, be thus generated? It has long been conjectured that epidemic fevers are produced by living animalculi floating in the air, but their death and decomposition in stagnant water, and the exhalation of poisonous gases from this source has, as far as I know, not been recognized, as a cause of our Autumnal fevers.

